# Nanoplastic incorporation into an organismal skeleton

**DOI:** 10.1038/s41598-022-18547-4

**Published:** 2022-08-30

**Authors:** Marlena Joppien, Hildegard Westphal, Viswasanthi Chandra, Marleen Stuhr, Steve S. Doo

**Affiliations:** 1 Geoecology and Carbonate Sedimentology Group, Leibniz Centre for Tropical Marine Research (ZMT), Bremen, Germany; 2 Physical Science and Engineering Division, King Abdullah University of Science and Technology (KAUST), Thuwal, Saudi Arabia; 3 Department of Geosciences, University of Bremen, Bremen, Germany

**Keywords:** Environmental impact, Sedimentology

## Abstract

Studies on the effects of global marine plastic pollution have largely focused on physiological responses of few organism groups (e.g., corals, fishes). Here, we report the first observation of polymer nanoparticles being incorporated into the calcite skeleton of a large benthic foraminifera (LBF), a significant contributor to global carbonate production. While previous work on LBF has documented selectivity in feeding behaviour and a high degree of specialization regarding skeletal formation, in this study, abundant cases of nanoplastic encrustation into the calcite tests were observed. Nanoplastic incorporation was associated with formation of new chambers, in conjunction with rapid nanoplastic ingestion and subsequent incomplete egestion. Microalgae presence in nanoplastic treatments significantly increased the initial feeding response after 1 day, but regardless of microalgae presence, nanoplastic ingestion was similar after 6 weeks of chronic exposure. While ~ 40% of ingesting LBF expelled all nanoplastics from their cytoplasm, nanoplastics were still attached to the test surface and subsequently encrusted by calcite. These findings highlight the need for further investigation regarding plastic pollution impacts on calcifying organisms, e.g., the function of LBF as potential plastic sinks and alterations in structural integrity of LBF tests that will likely have larger ecosystem-level impacts on sediment production.

## Introduction

Plastic pollution was first reported in the 1970s^1^ and has since then been documented in all marine habitats^2^. Microplastic particles (1 µm to 5 mm in size), in particular, are considered potential hazards to ecosystems, through ingestion by marine biota^3,4^, permeation of trophic levels^5,6^ and as vectors for pollution (e.g., toxic waste, heavy metals, pathogens)^7–9^. Subsequently, nanoplastics (≤ 1 μm^10^) have become an area of concern as these are even more prone to passive ingestion^11^. One of the largely unknown impacts of micro- and nanoplastics is their potential to affect protective skeletal structures of marine calcifying organisms. Only recently, skeletal encrustation of microplastics in scleractinian corals was observed^12,13^.

One group of important carbonate producers is that of the photosymbiotic large benthic foraminifera^14^ (LBF), which exhibit a mixotrophic lifestyle similar to scleractinian corals, and host a wide range of endosymbiotic algae (e.g., diatoms, dinoflagellates, red algae)^15^. Calcareous foraminifera are generally categorized as hyaline or porcelaneous, depending on their test structure^16,17^. These unicellular organisms contribute to about 5% of the worldwide shallow water carbonate production^18,19^ with locally much higher densities^19^. Due to vast numbers and high turnover in coastal ecosystems, LBF contribute greatly to shoreline and island formation and stabilization^14^. Additionally, the ability to evolve rapidly and fill ecological niches makes these organisms a valuable tool for paleoenvironmental interpretations and environmental monitoring^20,21^.

Previous studies on plastic impacts on foraminifera have shown the ingestion of plastic particles in varied size ranges (0.5 μm to 6 μm in diameter) with species-specific selectivity towards certain sizes^22,23^, and an active feeding preference of biofilm-coated microplastics was observed in the LBF *Amphistegina gibbosa*^24^. Stress reactions (i.e., accumulation of neutral lipids and enhanced reactive oxygen species production) were observed in *Ammonia parkinsonia* by the uptake of nanoplastic particles^25^. The incorporation of microplastics into the agglutinated tests of *Textularia bocki* has been documented, accompanied by oxidative stress and protein aggregation in the exposed foraminifera^23^. However, leachates from seawater-soaked polypropylene (typically containing bisphenol A, octylphenol and nonylphenol)^26^ were reported to have no significant effect on locomotion or metabolism of *Haynesina germanica*^27^.

Here, we document the effects of nanoplastics exposure (≤ 1 µm particles) on the LBF *Amphistegina* (*A. gibbosa* and *A. lobifera*) that offer insights into organismal ingestion and egestion mechanisms of nanoplastic. *Amphistegina* spp. are hyaline LBF that grow by forming new test chambers through biologically mediated mineralization. Formation of new test chambers in these organisms is initiated through a cytoplasmic extrusion of the LBF, providing the shape for the newly forming chamber and a thin primary organic layer as the base for CaCO_3_ precipitation^28,29^. During a secondary calcification stage, the chamber wall thickening, the majority of CaCO_3_ is precipitated^30^.

Like other photosymbiotic LBF, *Amphistegina* spp. (referred to as *Amphistegina* hereafter) exhibit a facultative heterotrophic mode, acquiring nutrients through both symbiont autotrophy and heterotrophic feeding^31–34^. As heterotrophic feeding is vital for the growth of these organisms^31^, organismal ingestion and egestion mechanisms of nanoplastics were assessed in food choices of: (1) nanoplastic particles only (fluorescent functionalized polystyrene; ‘nanoplastic-only treatment’), (2) a mixture of *Nannochloropsis* microalgae and nanoplastic particles (‘mixed treatment’), and (3) microalgae only (‘control treatment’). The size of nanoplastic particles and microalgae (~ 1 μm) was chosen to correspond to the size of microalgae naturally occurring in the marine environment, to minimize potential bias to size.

## Nanoplastic incorporation into skeletal structure of LBF

**Figure 1:**
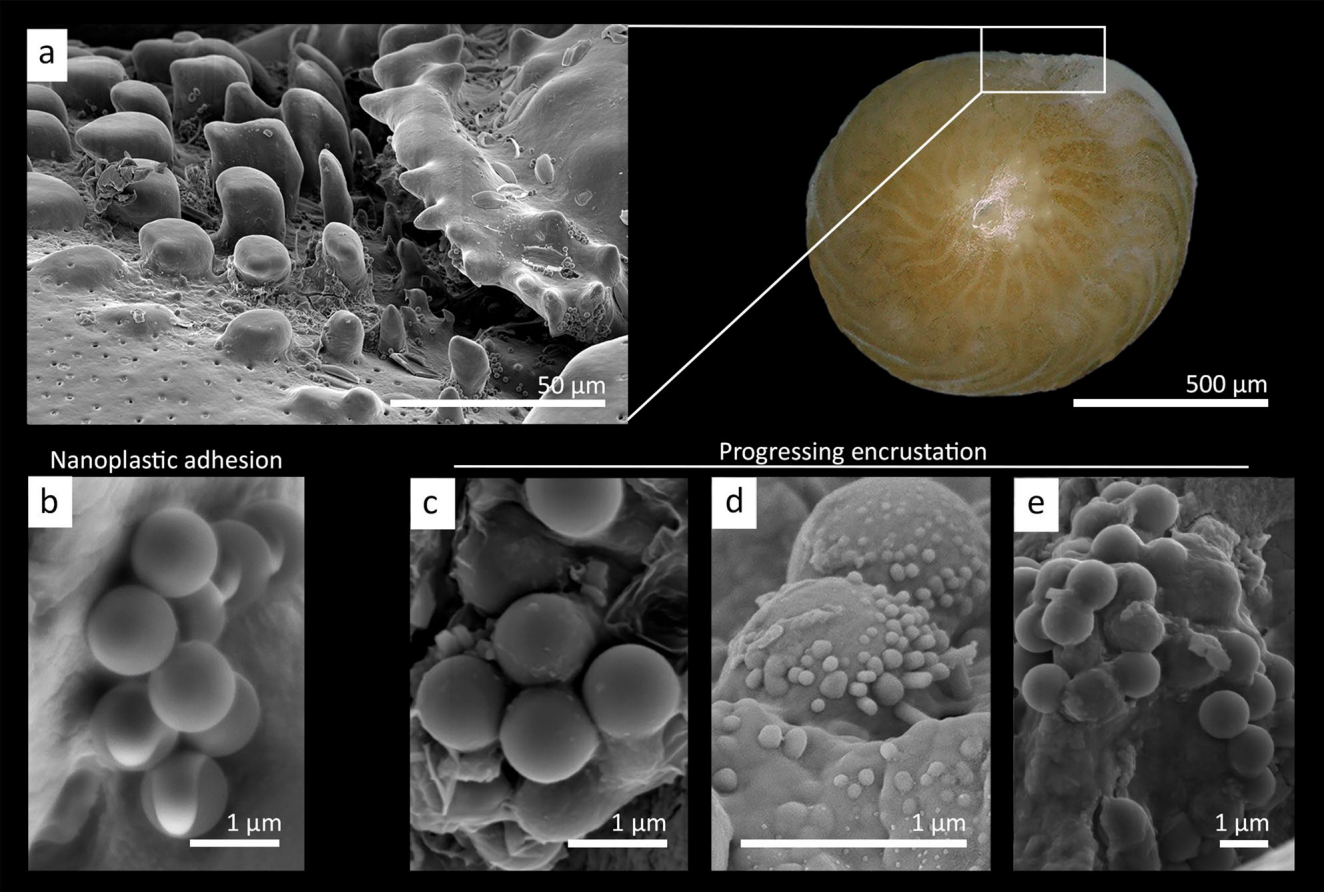
Photo of a living *A. gibbosa* (showing the ventral side) and SEM images of nanoplastic incorporation into the calcite test at the aperture. The nanoplastic particles can be identified as 1-μm spheres. (**a**) The ornamented area near the aperture of *Amphistegina* is shown, which is the opening to the newest chamber and the primary incorporation area. (**b**) Non-encrusted nanoplastic particles accumulated adjacent to the LBF test, which display no fusing to the test. In progressing encrustation phases, (**c**) calcite crystals are formed on nanoplastic particles fused to the test surface at the aperture. (**d**) Two particles in the process of incorporation, with crystals forming on top. (**e**) Several nanoplastic particles in varying stages of encrustation.

**Figure 2:**
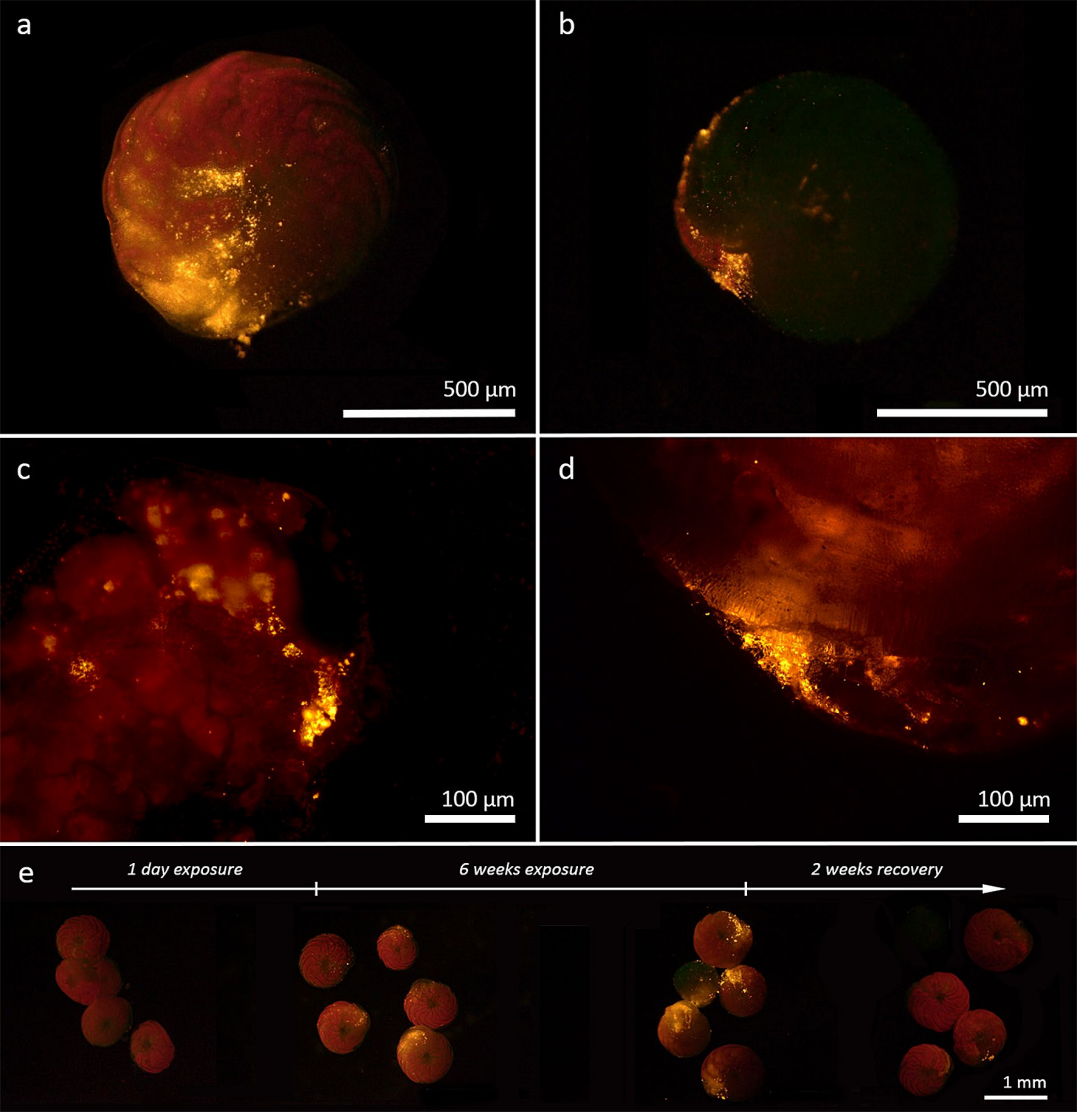
Examples showing cases of nanoplastic uptake and incorporation by fluorescence microscopy. (**a**) LBF seen here with ingested nanoplastic fluorescing yellow. (**b**) LBF with nanoplastic located only in the most recently formed chamber and at the aperture. Images (**c**) and (**d**) show sections of the foraminiferal tests with a view into the calcified test walls, providing closeups of the incorporation site. Pictures (**b**) and (**d**) show the same LBF individual from the ventral and dorsal side. (**e**) Timeline of nanoplastic ingestion in one exemplary replicate. Prior to nanoplastic exposure, five healthy LBF specimens show red fluorescence due to the autofluorescence of LBF symbionts. After 1 day of exposure, the uptake of yellow fluorescent nanoplastic is visible. Uptake of nanoplastic increased after 6 weeks of exposure and the loss of red fluorescence (resulting in a loss of symbiont fluorescence) is visible in one specimen. After the 2-week recovery period, four of the LBF had successfully egested all nanoplastic inside their cytoplasm, with one bleached during the egestion period (and thus not visible under fluorescent light). Only one of the specimens had nanoplastic remaining in its first chamber.

This study documents the first known instance of nanoplastic incorporation into the skeleton of a calcifying organism (Fig. 1). Ingestion responses were documented with fluorescence microscopy after 1-day and 6-week exposure to nanoplastic and egestion after an additional 2-week recovery (Fig. 2). Subsequently, skeletal incorporation was investigated using Scanning Electron Microscopy (SEM). At the test surface of newly formed chambers, nanoplastic particles appear to be encrusted by calcite, primarily observed close to ornamental spikes at the aperture (Fig. 1a). Encrusted nanoplastic particles formed dome-like structures often in accumulations of larger aggregates (> 5 particles) observed in various degrees of encrustation (Fig. 1c–e). As the LBF aperture is an area of active food uptake, the incorporation of nanoplastic into the test likely occurred in conjunction with ingestion and incomplete egestion processes. While previous studies of foraminifera^22,23,25^ have observed the uptake of nanoplastic particles, egestion of foreign particles is largely unknown. Following the 2-week recovery period, the percentage of LBF containing nanoplastic significantly decreased compared to 6-week exposure (F_2,95_ = 22.73, p < 0.001; Supplementary Table 2a). However, nearly half of the total LBF still contained nanoplastic particles (47.0 ± 5.5% LBF, n = 160). While LBF seemed to efficiently egest those particles from their inner cytoplasm, they appear to have stuck to the outer primary organic layer present at the sites of calcification^29,35,36^ and were thus passively incorporated in the calcite test.

The calcite overgrowth on nanoplastic particles appears as calcite crusts, likely precipitated in the process of chamber wall thickening or new chamber formation^35,37^ (Fig. 1e). Previous studies document *Amphistegina* calcification mechanisms through the initial formation of calcite spheres that subsequently are bound by an organic matrix (seen as organic filaments) to form a primary wall structure^35^. Although these calcite spheres are slightly larger^35^ (~ 2–3 μm) than the nanoplastic spheres used in this study, similar organic filaments were observed spanning across multiple nanoplastic particles. This indicates that nanoplastic spheres could be perceived by the LBF as potential ‘building-material’ for a newly forming chamber or during localized repair processes. The process of biomineralization has been previously described in detail for the rotaliid foraminifera *Ammonia beccarii*^38^. The calcification stages of *A. beccarii*, in particular the calcification between organic layers^38^, resembles structures seen at nanoplastic incorporation sites investigated here (Supplementary Figs. 3 and 4). Nanoplastic spheres could even present a possible nucleation site for crystal formation^39^, as previously seen with bacteria and diatoms^40,41^, thereby acting as catalysts in LBF chamber formation or localized repair.

## Ingestion patterns have no effect on skeletal incorporation

**Figure 3:**
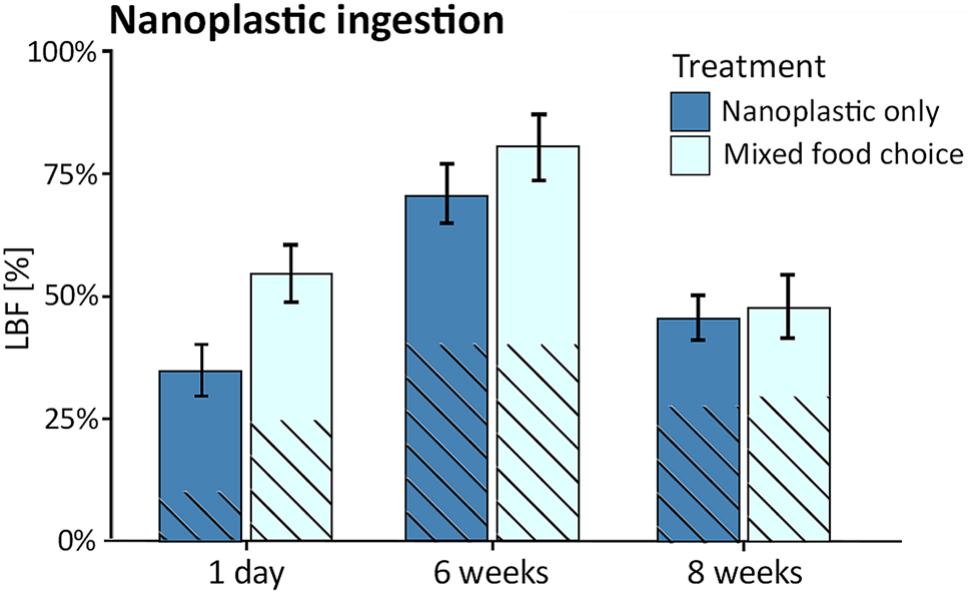
Ingestion occurrences on nanoplastics (% LBF; n = 80 LBF per treatment) observed in the nanoplastic-only treatment and in the mixed treatment. Hatched areas mark percentage of LBF with nanoplastic being located solely in the newest chamber. Ingestion occurrences are shown after 1 day of nanoplastic exposure, after 6 weeks of exposure, and after 8 weeks (6 weeks exposure + 2 weeks recovery). Error bars show standard error of total ingestion occurrences.

LBF generally utilize reticulopodia (a temporary extension of cytoplasm) to migrate food particles to aperture openings, subsequently engulfing particles via vacuolization^42^. LBF that were incubated in nanoplastic-only treatments exhibited a regulatory ability to avoid nanoplastic ingestion after 1 day of nanoplastic exposure (35.0 ± 5.1% LBF ingestion, n = 80, mean ± SE; Fig. 3). However, this potential regulatory ability against nanoplastic uptake was muted during a subsequent 6-week nanoplastic exposure, as seen in the high nanoplastic ingestion occurrences (71.0 ± 6.2% LBF ingestion; n = 80). In nanoplastic-only treatments, LBF specimens showed reduced growth (28.3 ± 8.9 μm growth in 8 weeks) compared to the mixed treatment (80.7 ± 12.9 μm growth) and the control treatment (96.6 ± 10.8 μm growth; F_2,95_ = 4.32, p = 0.02, Supplementary Table 1). As LBF growth rates and calcification partly depend on heterotrophic nutrient uptake of LBF^31^, the significantly decreased growth of LBF is likely due to providing nanoplastic without natural food sources, as nanoplastic presumably has no nutritional value.

The frequency of initial nanoplastic ingestion was significantly influenced by the presence of a natural food source. The presence of microalgae in the mixed treatment stimulated and significantly increased the initial feeding response during 1-day exposure (52.5 ± 5.8% LBF vs. 35.0 ± 5.1% in nanoplastic-only treatment; n = 80) compared to the nanoplastic-only treatment, and continued to increase during 6-weeks exposure (81.0 ± 6.7% LBF ingestion vs 71.0 ± 6.2% LBF ingestion; n = 80). This supports the importance of mimicking natural conditions in feeding experiments, showing that nanoplastic uptake in the presence of naturally occurring food sources is more common^24^. Although the ingestion of nanoplastic particles was initially increased in the presence of the natural food source, these ingestion patterns had no effect on egestion and skeletal incorporation, as the difference in egestion between the different treatments was insignificant (~ 40% egestion in both, nanoplastic-only and mixed treatment). Passive ingestion of nanoplastic through the pores was clearly distinguishable from active ingestion through the aperture and occurred exclusively in specimens that bleached during the experiment. Thus, we conclude that ingestion of nanoplastics is the result of active feeding. Bleaching of LBF holobionts remained low in all treatments (< 10%; Supplementary Table 1) and was not significantly altered by nanoplastic presence during the exposure period studied here (F_2,95_ = 1.11, p = 0.33, Supplementary Table 3). This contrasts previous studies that have shown that diatoms can be negatively impacted by microplastic exposure^43,44^.

## Implications for LBF and their sedimentological and ecological importance

In this study, we did not observe any deleterious effects of nanoplastics on the LBF calcareous tests (i.e., no test dissolution, breakages, major deformations). No effect on growth was observed when microalgal food sources were provided (i.e., in the mixed treatment; Supplementary Table 3). The frequent ingestion of nanoplastic particles in this study suggests an alternative mode of plastic invasion into marine food webs via unicellular organisms^45,46^, exacerbating bioaccumulation risks. Although egestion pathways reduce the residence time of microplastic inside the cytoplasm, egestion efforts without the benefit of nutrient uptake will potentially have negative impacts on foraminiferal energy budgets and consequentially on calcification and reproduction^31,33^.

Despite the partial egestion of ingested nanoplastic particles from the foraminiferal cytoplasm, particles were found to be abundantly incorporated into the test as a result of calcification. Skeletal incorporation of any type of foreign grains has previously not been documented for calcifying foraminifera, or any other calcite precipitating organisms. Further research employing sectioning methods like FIB-SEM for exploring the spatial relations of the skeletal carbonate and nanoplastics will allow for deeper insights into the encrustation. Additional work is also needed to ascertain further impacts of nanoplastics on LBF physiology and biomineralization. This is especially the case for longer-term or permanent nanoplastic exposure, as constant nanoplastic incorporation might promote skeletal malformations to a higher degree than relatively short-term exposure. One could hypothesize that the test structure and properties of hyaline foraminifera might change through the incorporation of nanoplastics, with potentially negative effects for the light conditions for symbiont photosynthesis, or test stability. Adhesion, ingestion and skeletal incorporation of nanoplastics could also become a potential sink for nanoplastic pollution, as previously hypothesized in the case of scleratinian corals^13,47,48^. Since LBF are essential components of tropical coral reef communities, the large-scale incorporation of nanoplastic into LBF tests as well as the potential consequences (e.g., test instability, toxicity) could influence ecosystem functions, such as carbonate production and coastline stability.

## Methods

### Collection and culture maintenance

We used specimens of *Amphistegina gibbosa* and *Amphistegina lobifera* from established long-term cultures in the marine experimental facility MAREE at the Leibniz Centre for Tropical Marine Research (ZMT) in Bremen, Germany. The collection of these LBF was described by Stuhr et al.^34,49^. The cultures have been maintained at the ZMT for 4–6 years at the time this experiment was conducted (summer 2021).

In culture, the LBF were kept in 500-ml containers filled with freshly made artificial seawater (made with *Red Sea Salt*) at a temperature of ~ 24 °C and light intensities of < 30 µmol m^−2^ s^−1^ (using a *JBL Solar Ultra MARIN Day 15000K* fluorescent light). It is assumed that the LBF used in this study are clonal progeny from the original cohort. During the culture period prior to the start of this experiment, the LBF used in this study were fed once a month with diluted *Nannochloropsis* algae concentrate (12 × 10^9^ cells ml^−1^, *BlueBioTech GmbH*, Germany). Culture maintenance is described further by Schmidt et al.^50^.

## Experimental setup

### Food choices

To understand the impact of nanoplastic presence on physiological performance, feeding behavior and calcification of LBF, two food sources were provided in this study. The first was sterilized *Nannochloropsis* algae (12 × 10^9^ cells ml^−1^, *BlueBioTech GmbH*, Germany), a natural food source the LBF regularly encounter. The algae stock solution was created freshly prior to each feeding session, by diluting 0.015 ml algae concentrate in 100 ml seawater (concentration of stock solution: 3.60 × 10^8^ cells ml^−1^). The stock solution was autoclaved to avoid algal blooms inside the experimental treatments. The second food source were nanoplastic particles (Polystyrene (PS) grains; *Fluoresbrite*^*®*^* Polychromatic Red Microspheres*, 1.0 μm, 4.55 × 10^10^ particles ml^−1^). A stock solution of the nanoplastic concentrate was created freshly before feeding, by diluting 0.1 ml concentrate in 5 ml seawater (concentration of stock solution: 4.55 × 10^9^ particles ml^−1^). Both food particles, algae and nanoplastic, were approximately in the same size range (~ 1 μm). Nanoplastic concentration in nanoplastic-only and mixed treatments was 1.8 × 10^7^ particles ml^−1^. *Nannochloropsis* concentration in mixed and control treatments was 1.8 × 10^4^ cells ml^−1^.

### Experimental exposure

For the duration of the experimental exposure to different food choices, LBF were kept in 12-well polystyrene plates (5 LBF per well; *CELLSTAR*). Three experimental treatments were set up: nanoplastic particles as the only food source (‘nanoplastic-only’; 9 × 10^7^ particles per 5-ml well), nanoplastic particles and algae (‘mixed’; 9 × 10^7^ nanoplastic particles and 9 × 10^4^ *Nannochloropsis* cells per 5-ml well), *Nannochloropsis* algae only (‘control’; 9 × 10^4^ cells per 5-ml well). Each treatment consisted of 80 LBF with 16 replicates. The concentration of *Nannochloropsis* cells inside the wells was relatively low to remain close to the culturing conditions. There was no direct comparison between the amount of algae uptake and nanoplastic uptake. Prior to the experiment start, the vitality of all LBF used in this experiment was assessed visually through symbiont coloring and symbiont fluorescence intensity, to assure the health of all LBF. The chosen LBF were then fed weekly with the respective food choices over a course of 6 weeks. For an additional 2 weeks of recovery period, nanoplastic was not added to allow for detection of egestion, however, microalgae were still provided.

### Evaluation of ingestion, vitality, and size

Every week, all LBF were rinsed individually with fresh seawater to avoid excessive accumulation of nanoplastic and algae inside the wells and on the LBF itself. This also assured that all nanoplastic that was covering only the outside test of the LBF would not be included in the ingestion identification. Pictures of the LBF were taken after 1 day, 6 weeks and 8 weeks (6 weeks exposure + 2 weeks recovery) to assess feeding response (ingestion of nanoplastic) and vitality (i.e., symbiont coloring and presence of symbiont fluorescence as an indicator for bleaching). Ingestion was assessed by counting the LBF individuals that had nanoplastic particles solely inside their first (newest) chamber and LBF that showed nanoplastic particles further inside their test. Sizes of all LBF individuals were monitored by measuring the diameter of the specimens prior to the experiment start, after 1 day, 6 weeks and 8 weeks on optical micrographs taken with the *Keyence Digital Microscope VHX-2000*. Fluorescence imaging was done with an additional fluorescence adapter (Filter set Cyan, Excitation 490–515 nm, *NIGHTSEA*, USA).

### Documentation of nanoplastic incorporation

Sections of individual LBF were prepared to detect the exact position of nanoplastic inside the LBF and account for potential incorporation of nanoplastic particles into the calcite test. High resolution fluorescence microscopy was done using a *Leica DM6000B* microscope. Potential nanoplastic incorporation sites were further analyzed with scanning electron microscopy (SEM; *Teneo Thermo Fisher Scientific*). Iridium coating was used to obtain SEM images at multiple magnifications up to 80.000×. For the highest magnification of 80.000×, 2 kV high voltage and a working distance of 2 mm was used. All other images were taken at 5 kV high voltage and working distances of 10 mm. As aggressive removal of organics might have damaged the nanoplastic beads, the methods chosen here leave the possibility that remains of extracellular polymeric substances (EPS) have stayed in the sample and might have influenced the surface.

EDX analysis was conducted to confirm the presence of CaCO_3_ encrustations (Supplementary Fig. 5). For the EDX an energy of 10 keV was applied, resulting in a penetration depth which is larger than particle size. Thus, the interpretation of the EDX data is limited as the foraminiferal shell material underlying the nanoplastics particle could have been excited. Nevertheless, the sharp peaks argue against a pure substrate signal. Evidence of nanoplastic incorporation is mainly based on morphological information acquired from SEM.

### Data analysis

The number of LBF that ingested nanoplastic was counted within treatments and converted to percentages (% LBF per treatment that ingested nanoplastic). The same was done for the number of bleached LBF (as an indicator for vitality). A two-way ANOVA was conducted to detect significant differences in feeding responses (total ingestion of nanoplastic and nanoplastic inside the first chamber only), vitality and organism size between treatments. Treatment (nanoplastic-only, mixed, control) and duration (1 day = T1, 6 weeks = T2, 8 weeks = T3) were assigned as fixed factors. Prior to the ANOVA analyses, all data were tested for normality (Shapiro–Wilk) and homogeneity of variance (Levene’s test). Normality and homogeneity assumptions (p > 0.05) were met in size and bleaching measurements, however, the nanoplastic feeding observations were not normally distributed. We still proceeded with the analysis due to the robust nature of ANOVA tests. All ANOVA analyses were performed in R Version 4.0.2 using the R stats package^51^ and vegan package^52^.

## Supplementary Information

Supplementary Information 1.

Supplementary Information 2.

## Data Availability

The data generated or analyzed during this study are included in the supplementary information files of this published article.

## References

[1] Carpenter EJ, Anderson SJ, Harvey GR, Miklas HP, Peck BB (1972). Polystyrene spherules in coastal waters. Science.

[2] Fischer V, Elsner NO, Brenke N, Schwabe E, Brandt A (2015). Plastic pollution of the Kuril-Kamchatka Trench area (NW pacific). Deep Sea Res. Part II.

[3] GESAMP. *Sources, fate and effects of microplastics in the marine environment: A global assessment * (ed Kershaw, P. J.) 96 (2015).

[4] SAPEA. *A Scientific Perspective on Microplastics in Nature and Society*. 10.26356/microplastics (2019).

[5] Andrady AL (2011). Microplastics in the marine environment. Mar. Pollut. Bull..

[6] Lusher A, Bergmann M, Gutow L, Klages M (2015). Microplastics in the marine environment: Distribution, interactions and effects. Marine Anthropogenic Litter.

[7] Rios LM, Moore C, Jones PR (2007). Persistent organic pollutants carried by synthetic polymers in the ocean environment. Mar. Pollut. Bull..

[8] Teuten EL, Rowland SJ, Galloway TS, Thompson RC (2007). Potential for plastics to transport hydrophobic contaminants. Environ. Sci. Technol..

[9] Viršek MK, Lovšin MN, Koren Š, Kržan A, Peterlin M (2017). Microplastics as a vector for the transport of the bacterial fish pathogen species *Aeromonas salmonicida*. Mar. Pollut. Bull..

[10] Gigault J (2018). Current opinion: What is a nanoplastic?. Environ. Pollut..

[11] Shen M (2019). Recent advances in toxicological research of nanoplastics in the environment: A review. Environ. Pollut..

[12] Hierl F, Wu HC, Westphal H (2021). Scleractinian corals incorporate microplastic particles: Identification from a laboratory study. Environ. Sci. Pollut. Res..

[13] Reichert J (2021). Reef-building corals act as long-term sink for microplastic. Glob. Change Biol..

[14] Narayan GR (2021). Response of large benthic foraminifera to climate and local changes: Implications for future carbonate production. Sedimentology.

[15] Hottinger L (1982). Larger foraminifera, giant cells with a historical background. Naturwissenschaften.

[16] Dubicka, Z. Chamber arrangement versus wall structure in the high-rank phylogenetic classification of Foraminifera. *APP* **64** (2019).

[17] Pawlowski J, Holzmann M, Tyszka J (2013). New supraordinal classification of Foraminifera: Molecules meet morphology. Mar. Micropaleontol..

[18] Langer MR (2008). Assessing the contribution of foraminiferan protists to global ocean carbonate production. J. Eukaryot. Microbiol..

[19] Doo SS, Hamylton S, Finfer J, Byrne M (2017). Spatial and temporal variation in reef-scale carbonate storage of large benthic foraminifera: A case study on One Tree Reef. Coral Reefs.

[20] Hallock P, Lidz BH, Cockey-Burkhard EM, Donnelly KB, Melzian BD, Engle V, McAlister M, Sandhu S, Eads LK (2003). Foraminifera as bioindicators in coral reef assessment and monitoring: The Foram index. Coastal Monitoring Through Partnerships.

[21] Boudagher-Fadel MK (2018). Evolution and Geological Significance of Larger Benthic Foraminifera.

[22] Grefstad, A. I., Hylland, K., Alve, E. & Bour, A. C. Marine benthic foraminifera and microplastics—Accumulation and effects following short- and long-term exposure (2019).

[23] Birarda G (2021). Plastics, (bio)polymers and their apparent biogeochemical cycle: An infrared spectroscopy study on foraminifera. Environ. Pollut..

[24] Joppien M, Westphal H, Stuhr M, Doo SS (2022). Microplastics alter feeding strategies of a coral reef organism. Limnol. Oceanogr. Lett..

[25] Ciacci C (2019). Nanoparticle–biological interactions in a marine benthic foraminifer. Sci. Rep..

[26] Hermabessiere L (2017). Occurrence and effects of plastic additives on marine environments and organisms: A review. Chemosphere.

[27] Langlet D, Bouchet VMP, Delaeter C, Seuront L (2020). Motion behavior and metabolic response to microplastic leachates in the benthic foraminifera *Haynesina germanica*. J. Exp. Mar. Biol. Ecol..

[28] Towe KM, Cifelli R (1967). Wall ultrastructure in the calcareous foraminifera: Crystallographic aspects and a model for calcification. J. Paleontol..

[29] de Nooijer LJ, Spero HJ, Erez J, Bijma J, Reichart GJ (2014). Biomineralization in perforate foraminifera. Earth Sci. Rev..

[30] Nagai Y, Uematsu K, Wani R, Toyofuku T (2018). Reading the fine print: Ultra-microstructures of foraminiferal calcification revealed using focused ion beam microscopy. Front. Mar. Sci..

[31] Hallock P (1981). Algal symbiosis: A mathematical analysis. Mar. Biol..

[32] Lee, J. J., Erez, J., Ter Kuile, B. H., Lagziel, A. & Burgos, S. Feeding rates of two species of larger foraminifera *Amphistegina**lobifera* and *Amphisorus**hemprichii*, from the Gulf of Eilat (Red Sea). 41 (1988).

[33] Ter Kuile BH, Erez J (1991). Carbon budgets for two species of benthonic symbiont-bearing foraminifera. Biol. Bull..

[34] Stuhr M (2018). Disentangling thermal stress responses in a reef-calcifier and its photosymbionts by shotgun proteomics. Sci. Rep..

[35] Bentov S, Erez J (2005). Novel observations on biomineralization processes in foraminifera and implications for Mg/Ca ratio in the shells. Geology.

[36] Bentov S, Brownlee C, Erez J (2009). The role of seawater endocytosis in the biomineralization process in calcareous foraminifera. Proc. Natl. Acad. Sci..

[37] de Nooijer LJ, Toyofuku T, Kitazato H (2009). Foraminifera promote calcification by elevating their intracellular pH. Proc. Natl. Acad. Sci..

[38] Nagai Y (2018). Weaving of biomineralization framework in rotaliid foraminifera: Implications for paleoceanographic proxies. Biogeosciences.

[39] Mahadevan G, Ruifan Q, Hian Jane YH, Valiyaveettil S (2021). Effect of polymer nano- and microparticles on calcium carbonate crystallization. ACS Omega.

[40] Emeis K-C, Richnow H-H, Kempe S (1987). Travertine formation in Plitvice National Park, Yugoslavia: Chemical versus biological control. Sedimentology.

[41] Knorre H, Krumbein WE, Riding RE, Awramik SM (2000). Bacterial calcification. Microbial Sediments.

[42] Bowser SS, McGee-Russell SM, Rieder CL (1985). Digestion of prey in foraminifera is not anomalous: A correlation of light microscopic, cytochemical, and hvem technics to study phagotrophy in two allogromiids. Tissue Cell.

[43] Wang J (2016). Effects of plastic film residues on occurrence of phthalates and microbial activity in soils. Chemosphere.

[44] Guo Y (2020). Effects of microplastics on growth, phenanthrene stress, and lipid accumulation in a diatom, *Phaeodactylum tricornutum*. Environ. Pollut..

[45] Setälä O, Fleming-Lehtinen V, Lehtiniemi M (2014). Ingestion and transfer of microplastics in the planktonic food web. Environ. Pollut..

[46] Santos RG, Machovsky-Capuska GE, Andrades R (2021). Plastic ingestion as an evolutionary trap: Toward a holistic understanding. Science.

[47] Martin C, Corona E, Mahadik GA, Duarte CM (2019). Adhesion to coral surface as a potential sink for marine microplastics. Environ. Pollut..

[48] Corona E, Martin C, Marasco R, Duarte CM (2020). Passive and active removal of marine microplastics by a mushroom coral (*Danafungia*
*scruposa*). Front. Mar. Sci..

[49] Stuhr M (2021). Divergent proteomic responses offer insights into resistant physiological responses of a reef-foraminifera to climate change scenarios. Oceans.

[50] Schmidt C (2015). Recent invasion of the symbiont-bearing foraminifera pararotalia into the eastern Mediterranean facilitated by the ongoing warming trend. PLoS One.

[51] R Core Team (2020). R: A Language and Environment for Statistical Computing.

[52] Oksanen, J. *et al. vegan: Community Ecology Package* (2020).

