# Supplementary material for: Nanoplastic incorporation into an organismal skeleton
Source: Sci Rep. 2022 Aug 30;12:14771. doi: 10.1038/s41598-022-18547-4 (PMC9427768; doi:10.1038/s41598-022-18547-4)
Supplement: Supplementary file 2 — Supplementary Information 2. [file 41598_2022_18547_MOESM2_ESM.docx]

# **Supplementary material for:**

# **Nanoplastic incorporation into an organismal skeleton**

Marlena Joppien^1,2,3^, Hildegard Westphal^1,2,3^, Viswasanthi Chandra^2^, Marleen Stuhr^1^, Steve S. Doo^1,2^

^1^Geoecology and Carbonate Sedimentology Group, Leibniz Centre for Tropical Marine Research (ZMT), Bremen, Germany
^2^King Abdullah University of Science and Technology (KAUST), Thuwal, Saudi Arabia

^3^Department of Geosciences, University of Bremen, Bremen, Germany

 Corresponding author: Marlena Joppien, marlena.joppien@kaust.edu.sa


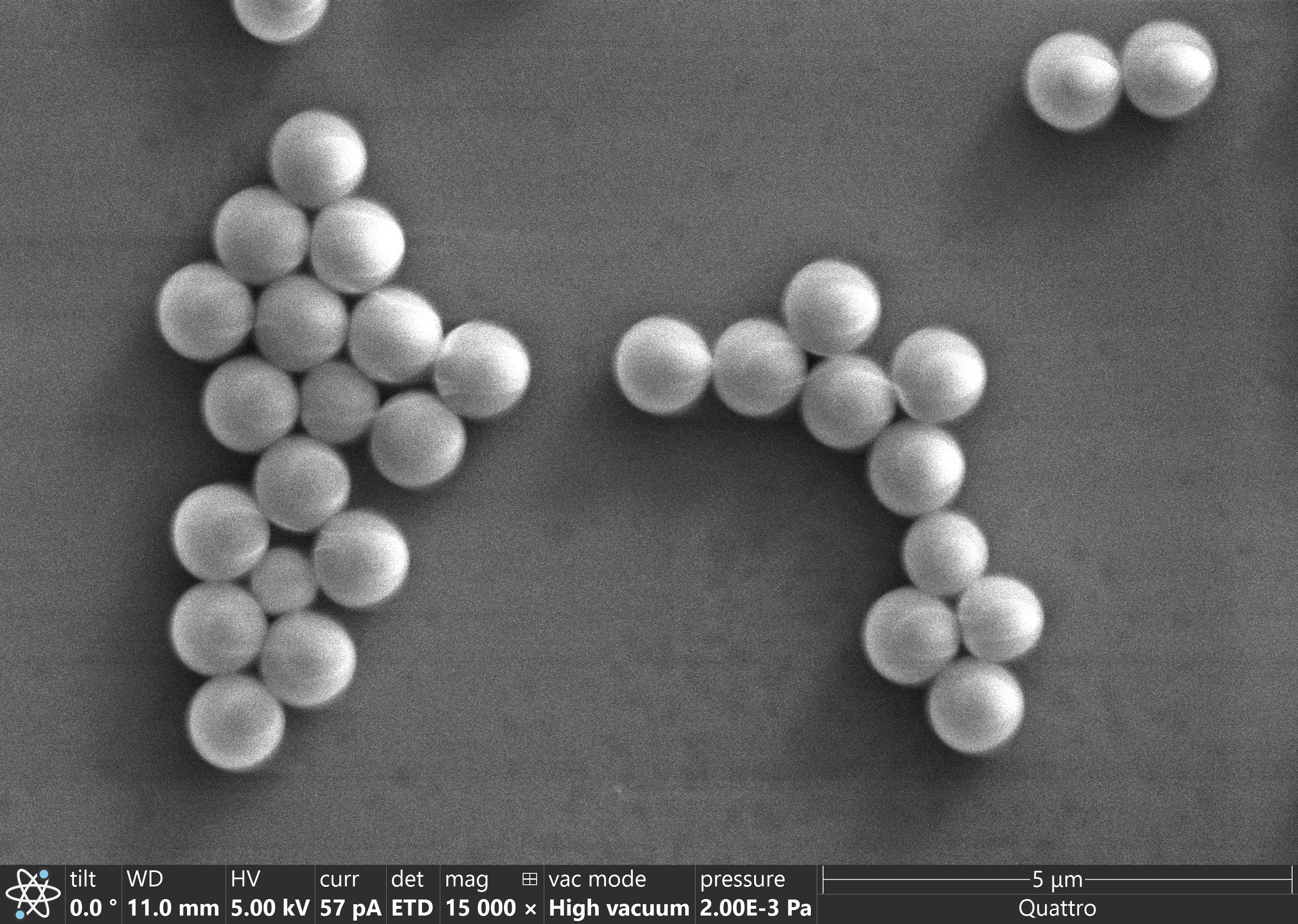


**Supplementary Figure 1** Control picture of nanoplastic particles taken with SEM. Nanoplastic particles here were imaged separate from the experimental treatments, to ensure correct identification of particles.


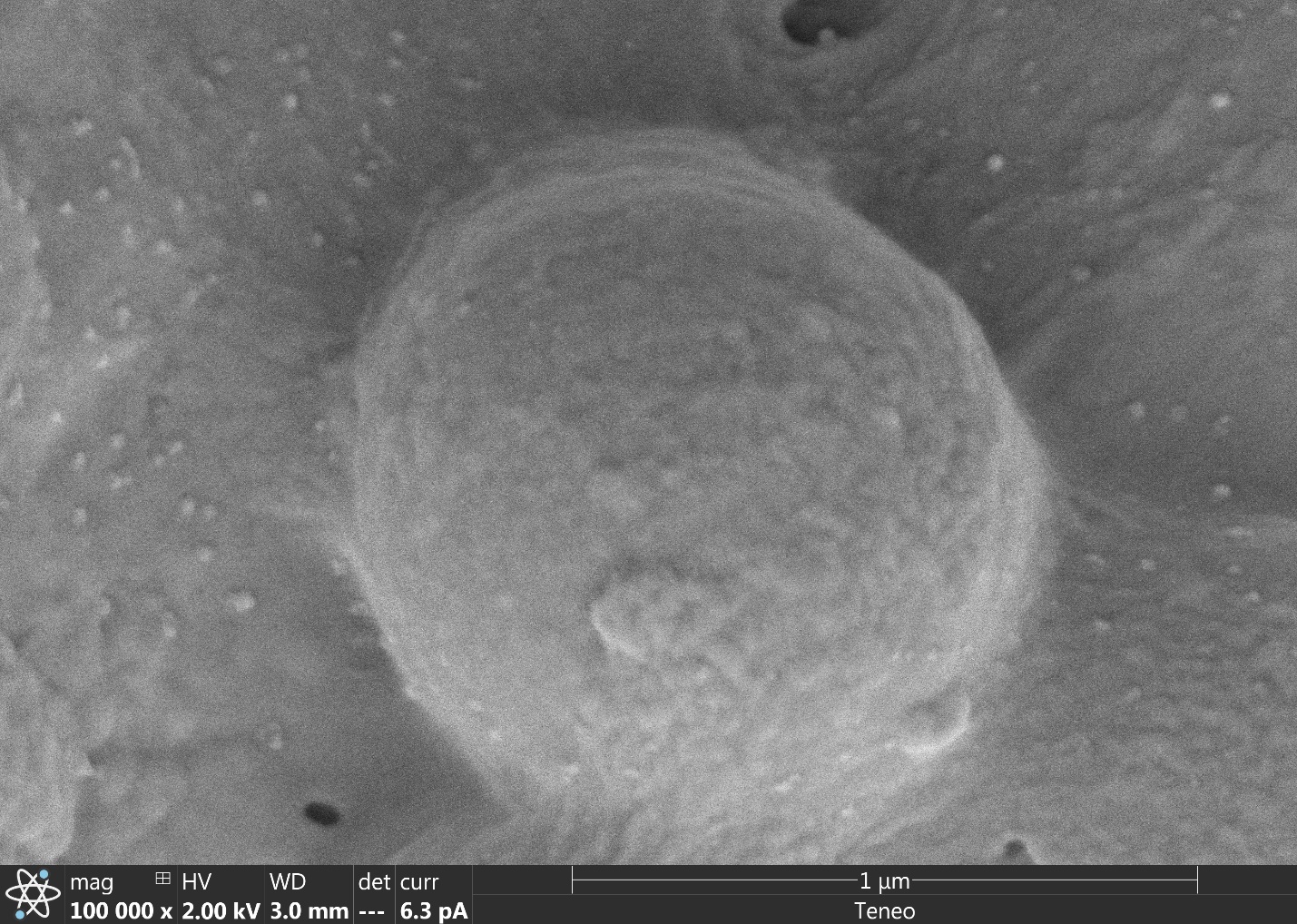


**Supplementary Figure 2** A fully encrusted particle on the test surface.


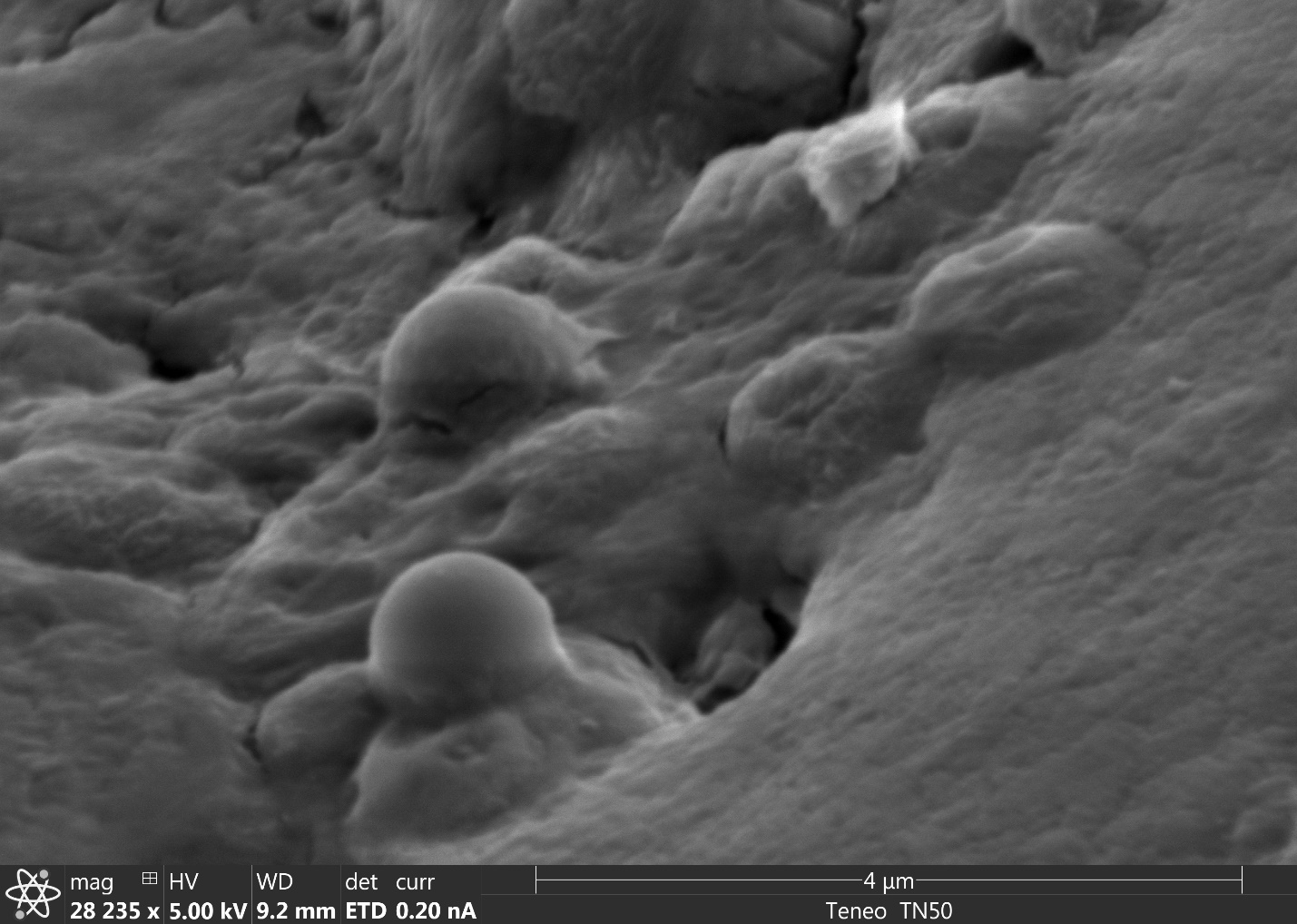


**Supplementary Figure 3** Several incorporated particles at the test surface. The nanoplastic particles are seen in different stages of encrustation. Circular patterns on the surface could potentially signal fully incorporated nanoplastic spheres.


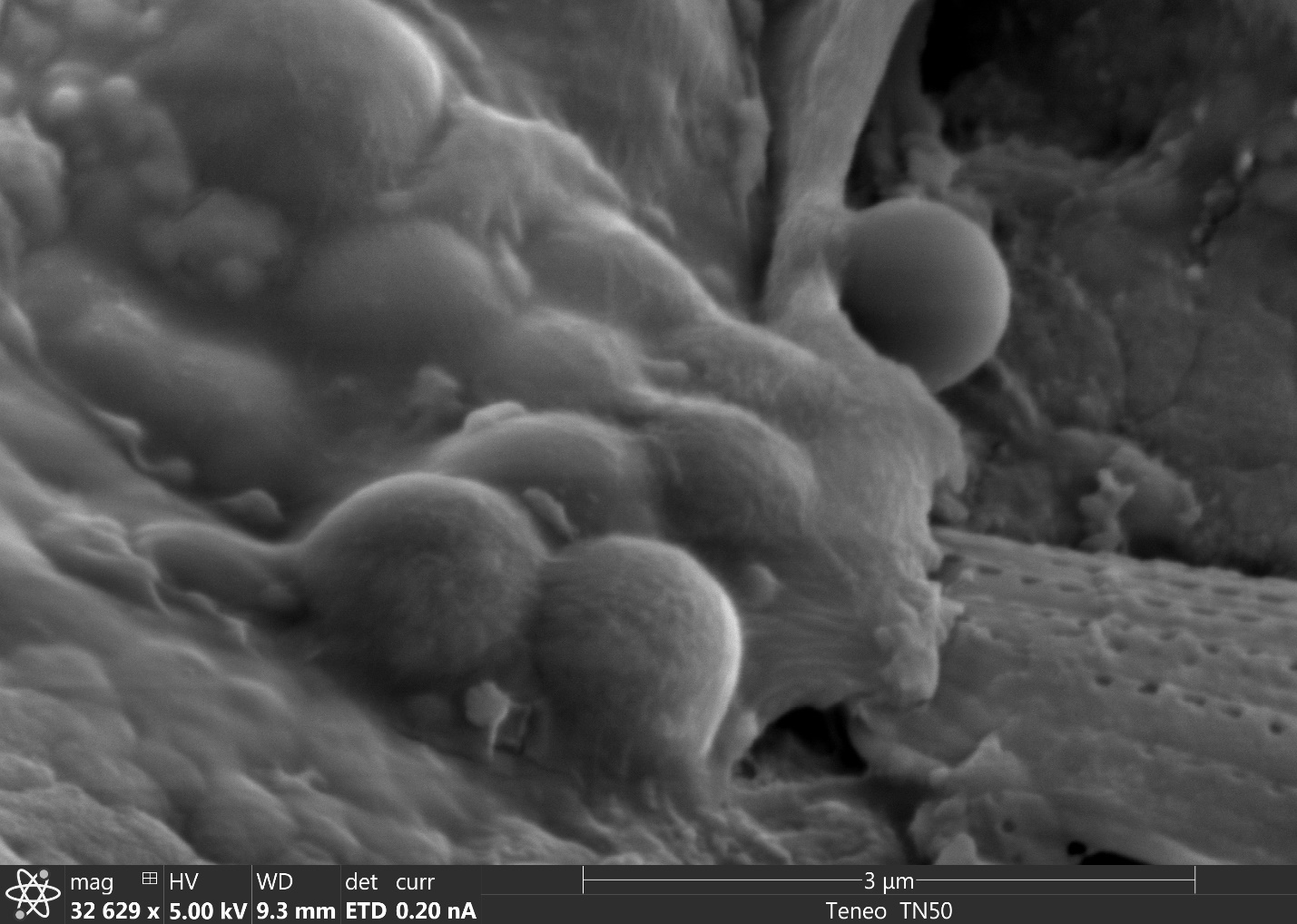


**Supplementary Figure 4** Nanoplastic particles on the test surface which are potentially encrusted or coved by an organic layer.


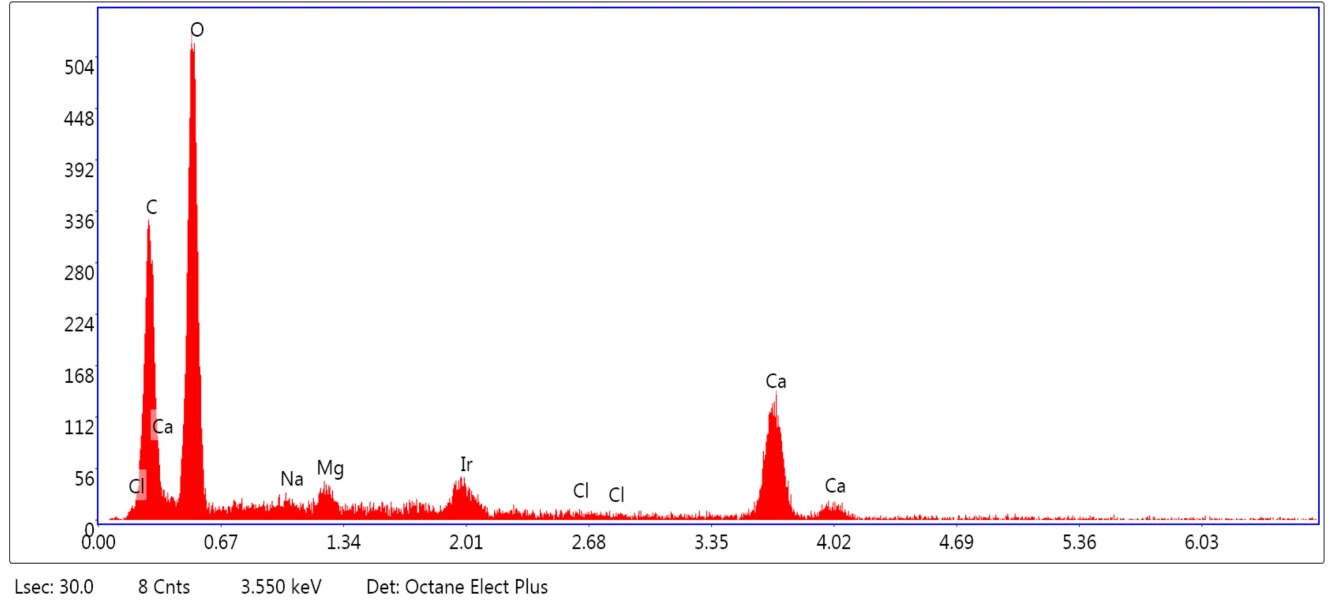


**Supplementary Figure 5** EDX results taken at the location of an encrusted particle.

**Supplementary Table 1** Results for (a) LBF size and (b) bleaching in nanoplastic-only treatments, mixed food choice and control (microalgae-only) at measurement points T1 (1 day), T2 (6 weeks) and T3 (8 weeks). LBF size over the course of the experiment might have been influenced by species specific growth rate differences in the two *Amphistegina* species used here.

| 1. **Ø Size ± SE [μm]** | | | | 1. **Ø Bleached LBF ± SE [%]** | | | | | |
| --- | --- | --- | --- | --- | --- | --- | --- | --- | --- |
|  | *nanoplastic -only* | *mixed* | *control* |  | *nanoplastic -only* | *mixed* | | | *control* |
| *T1* | 853.1 ± 18.3 | 840.1 ± 18.4 | 827.6 ± 19.7 | *T1* | 2.5 ± 1.7 | 3.8 ± 2.0 | | | 0 |
| *T2* | 887.1 ± 17.3 | 927.1 ± 19.8 | 907.1 ± 20.8 | *T2* | 7.5± 3.0 | 8.8 ± 3.5 | | | 4.0 ± 2.6 |
| *T3* | 883.4 ± 17.0 | 922.2 ± 19.7 | 912.5 ± 21.8 | *T3* | 9.0 ± 3.5 | 9.0 ± 3.5 | | | 8.0 ± 3.0 |
| ***Growth*** | 28.3 ± 8.9 | 80.7 ± 12.9 | 80.9 ± 10.8 |  |  | |  |  | |

**Supplementary Table 2** Results of two-way ANOVA, analysing the effect of treatments (nanoplastic-only [NP] vs. microalgae and nanoplastic [mixed]; n = 80) and experiment duration (1 day [T1] vs. 6 weeks [T2] vs. 8 weeks [T3]) on ingestion occurrences. Ingestion occurrences were analysed (a) in total and (b) with nanoplastic only in the first chamber.

| 1. **Ingestion of nanoplastic, total occurrences** | | | | | | |
| --- | --- | --- | --- | --- | --- | --- |
| *Source* | *Df* | *SS* | *MS* | *F* | *p* | *Tukey* |
| Duration | 2 | 2.77 | 1.385 | 22.726 | **1.02E-08** | **T1-T2, T2-T3** |
| Treatment | 1 | 0.602 | 0.6017 | 9.872 | **0.00227** | **[NP]-[mixed]** |
| Duration:Treatment | 2 | 0.043 | 0.0217 | 0.356 | 0.70179 |  |
| Residuals | 90 | 5.485 | 0.0609 |  |  |  |
| Total | 95 | 8.9 | 2.0693 |  |  |  |
|  | | | | | | |
| 1. **Nanoplastic located only in newest chamber** | | | | | | |
| *Source* | *Df* | *SS* | *MS* | *F* | *p* | *Tukey* |
| Duration | 2 | 0.951 | 0.4754 | 10.442 | **8.35E-05** | **T1-T2** |
| Treatment | 1 | 0.05 | 0.0504 | 1.107 | 0.295 |  |
| Duration:Treatment | 2 | 0.081 | 0.0404 | 0.888 | 0.415 |  |
| Residuals | 90 | 4.097 | 0.0455 |  |  |  |
| Total | 95 | 5.179 | 0.6117 |  |  |  |
|  | | | | | | |

**Supplementary Table 3** Results of two-way ANOVA, analysing the effect of treatments (nanoplastic only [NP] vs. microalgae and nanoplastic [mixed] vs. control; n = 80) and experiment duration (1 day [T1] vs. 6 weeks [T2] vs. 8 weeks [T3]) on (a) LBF growth and (b) bleaching.

| 1. **Growth of LBF** | | | | | | |
| --- | --- | --- | --- | --- | --- | --- |
| *Source* | *Df* | *SS* | *MS* | *F* | *p* | *Tukey* |
| Duration | 1 | 453 | 453 | 0.077 | 0.7824 |  |
| Treatment | 2 | 51029 | 25515 | 4.318 | **0.0162** | **[mixed]-[NP], [control]-[NP]** |
| Duration:Treatment | 2 | 553 | 277 | 0.047 | 0.9543 |  |
| Residuals | 90 | 531758 | 5908 |  |  |  |
| Total | 95 | 583793 | 32153 |  |  |  |
| 1. **Percentage of bleached LBF over time** | | | | | | |
| *Source* | *Df* | *SS* | *MS* | *F* | *p* | *Tukey* |
| Duration | 2 | 0.1006 | 0.05028 | 3.862 | **0.0234** | **T1-T3** |
| Treatment | 2 | 0.0289 | 0.01444 | 1.11 | 0.3327 |  |
| Duration:Treatment | 4 | 0.0061 | 0.00153 | 0.117 | 0.9762 |  |
| Residuals | 135 | 1.7575 | 0.01302 |  |  |  |
| Total | 143 | 1.8931 | 0.07927 |  |  |  |
